# Role of Natural Stilbenes in the Prevention of Cancer

**DOI:** 10.1155/2016/3128951

**Published:** 2015-12-21

**Authors:** J. Antoni Sirerol, María L. Rodríguez, Salvador Mena, Miguel A. Asensi, José M. Estrela, Angel L. Ortega

**Affiliations:** Department of Physiology, Faculty of Pharmacy, University of Valencia, Avenida Vicente Andrés Estellés s/n, Burjassot 46100, Valencia, Spain

## Abstract

Natural stilbenes are an important group of nonflavonoid phytochemicals of polyphenolic structure characterized by the presence of a 1,2-diphenylethylene nucleus. Stilbenes have an extraordinary potential for the prevention and treatment of different diseases, including cancer, due to their antioxidant, cell death activation, and anti-inflammatory properties which associate with low toxicity under *in vivo* conditions. This review aims to discuss various approaches related to their mechanisms of action, pharmacological activities in animal models and humans, and potential chemoprevention in clinical studies. The biological activity of natural stilbenes is still incompletely understood. Furthermore, after administration to animals or humans, these molecules are rapidly metabolized. Thus pharmacokinetics and/or activities of the natural structures and their metabolites may be very different. Novel drug formulations have been postulated in order to improve stability and bioavailability, to minimize side effects, and to facilitate interaction with their domains in target proteins. These pharmacological improvements should lead stilbenes to become effective candidates as anticancer drugs.

## 1. Introduction

Despite the fact that the total European population comprises just one-ninth of the world's population, the percentage of the global burden of cancer in Europe is of approximately 25% [1]. Recent epidemiological research estimates that approx. 1,323,000 and 585,000 deaths were caused by cancer in the European Union and the United States, respectively, in 2014 [2, 3]. At the beginning of 21st century cancer was the second cause of death only preceded by cardiovascular diseases and followed by diseases derived from complications associated with diabetes and chronic respiratory diseases [4]. This tendency has been changing with time and nowadays cancer exceeds the cardiovascular diseases mortality rate in some advanced countries, possibly due to improvements in patient care, more effective therapies, and awareness of the population to acquire healthier life style [3, 5, 6]. In consequence, considerable attention has been focused on chemoprevention as an alternative approach to the control of cancer.

Multiple evidences suggest that oxidative stress induced by reactive oxygen species (ROS) is closely related to multistage carcinogenesis [7]. ROS are the most abundant free radicals in cells and have been related with a number of tissue/organ injuries. Oxidative stress is caused by an imbalance between ROS production and the biological system's ability to neutralize or remove ROS by specific scavengers and the antioxidant enzymatic machinery. Thus, oxidative stress can cause protein, lipid, and DNA damage and thereby modulate/trigger initiation, promotion, and progression of cancer [8]. In this sense, antioxidants are defined as compounds that can delay, inhibit, or prevent the oxidative damage by scavenging free radicals and diminishing oxidative stress [9].

**Figure 1:**
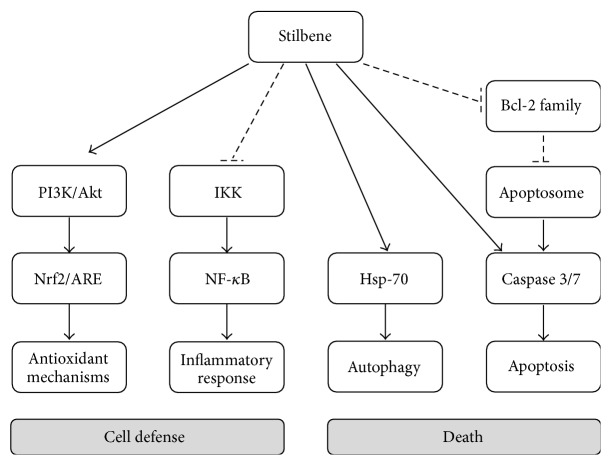
Anticarcinogenic mechanisms induced by major stilbenes.

During the last 20 years the interest in phytochemicals of polyphenolic structure has grown considerably. Natural polyphenols are plant secondary metabolites generated through the shikimate-derived phenylpropanoid and/or the polyketide pathway(s), with two or more phenolic rings, and being devoid of any nitrogen-based functional group in their basic structure [10]. They are produced by plants to protect themselves against stressing situations such as excessive ultraviolet (UV) irradiation, heat exposition, insects attacks, and fungus or bacterial infections [10]. Over 8,000 different phenolic compounds have been identified in the plant kingdom. Natural polyphenols are abundant in fruits, vegetables, whole grains, and foods and beverages derived from them, such as chocolate, wine, olive oil, or tea, thus becoming the most important among all phytochemicals present in the human diet [11]. Natural polyphenols have received increasing attention due to their potent antioxidant properties and their marked effects in the prevention of various oxidative stress associated diseases such as cancer [12, 13]. Indeed, potential anticancer properties have been suggested for various polyphenols, including, for example, green tea polyphenols, grape seed proanthocyanidins, resveratrol, silymarin, curcumin, quercetin, luteolin, and genistein [14, 15]. Although the chemopreventive effects of natural polyphenols are mainly due to their antioxidant activity, mechanistic studies suggest that, in addition, they have multiple intracellular targets (Figure 1) [7].

Natural stilbenes are a group of polyphenols characterized by the presence of a 1,2-diphenylethylene nucleus [16, 17]. There are more than 400 natural stilbenes [16], however they are present in a limited and heterogeneous group of plant families since the key enzyme involved in stilbene biosynthesis, stilbene synthase, is not ubiquitously expressed [17]. Since the original research by Jang et al. where a stilbene, resveratrol (Resv), was shown as a potent chemopreventive agent [18], these compounds have awakened the interest of the scientific community involved in anticancer drug development.

This review will focus on stilbenes and their potential as antioxidants and chemopreventive agents, thus including their molecular targets and signaling pathways; evidences from clinical trials for its toxicity, bioavailability, and benefit in humans; and biological improvements based on the development of analogs.

## 2. Cancer Chemopreventive Role of Natural Stilbenes

Cancer development is a progressive multistep process started with initial driver mutations (initiation) and followed by promotion and progression that ultimately lead to malignancy. Administration and consumption of agents to prevent, inhibit, or delay carcinogenesis are gathered in the global concept of chemoprevention [7, 19]. Stilbenes have shown ability to reduce the incidence of tumorigenesis by interfering with molecular events at all steps, that is, initiation, promotion, and progression stages of carcinogenesis. The limited distribution of the stilbenes in the plant kingdom led anticancer studies to focus on a reduced number of compounds [20]. With similarities and particularities, the number of targets and mechanisms where they are involved paved the way to their protective or therapeutic effects against cancer.

### 2.1. Resveratrol

Resv (3,4′,5-trihydroxy stilbene) was originally identified as a phytoalexin by Langcake and Pryce [21]. This natural stilbene has been found in at least 185 plant species [17] and is present in foods and beverages derived from them such as, for example, mulberries, peanuts, grapes, and red wine [18]. Its potential anticancer activity was originally reported by Jang et al. [18] and more than 2,000 references may be found in PubMed crossing Resv and cancer, thus showing the great interests in their chemopreventive and chemotherapeutic properties. In fact, Resv has undergone* in vitro* and* in vivo* carcinogenesis assays for many types of cancers, that is, breast [22], lung [23], colon [24], skin (nonmelanoma skin cancer and melanoma) [25], prostate [26], ovarian [27], liver [28], oral cavities [29], thyroid [30], and leukemia [31].

The chemopreventive properties of Resv have been associated with its antioxidant activity since it was first published that its anticancer activity, affecting all steps in the carcinogenesis process, was linked to the inhibition of cyclooxygenase 2 (COX-2) [18].

Up to now three different COX isoforms have been described: COX-1, expressed in normal tissue, participating in tissue homeostasis; COX-2, overexpressed in case of inflammation or neoplasia development; and COX-3, a variant of COX-1 [32]. The important role of COX-2 in the progression of tumorigenesis is supported by studies that show an elevated level of the enzyme in premalignant and malignant tissue, which is accompanied by a decrease in the rate of survival of cancer patients [33, 34] and is a bad prognostic factor [35, 36]. Clinical trials have shown that COX-2 inhibitors may be a good strategy to prevent the development of colonic adenomas and potentially carcinomas [32]. However, the clinical efficacy of COX-2 inhibitors in the prevention of cancer has been challenged due to higher cardiovascular risks [37]. In this scenario, the use of natural compounds without toxic effects and demonstrated efficacy as potential COX-2 inhibitors, such as Resv, is of particular interest.

It has also been described that prostaglandins, produced by COX activity, are able to enhance cancer development and progression acting as tumor promoters or carcinogens [36, 38]. In fact, an increase in prostaglandin synthesis has important effects on carcinogen metabolism, tumor cell proliferation, and metastatic potential [39, 40] and may affect tumor growth in both humans and experimental animals. Thus inhibition of prostaglandin synthesis has been investigated to prevent tumor development [38, 40, 41].

Different authors have confirmed that Resv inhibits COX-2 expression and decreases prostaglandin E2 (PGE(2)) production. In this regard, Cianciulli et al. described that Resv downregulates COX-2 and PGE(2) in a concentration dependent fashion in the human intestinal cell line Caco-2 treated with lipopolysaccharide and that this mechanism may be related to NF-*κ*B inhibition [42]. NF-*κ*B is an inducible transcription factor strongly linked to inflammatory and immune responses and associated with oncogenesis [43]. Different stimuli may activate the release and translocation of NF-*κ*B to the nucleus (e.g., those activating some membrane receptors (B cell receptor or tumor necrosis factor receptors) or several extracellular stimuli (inflammatory cytokines, viral and bacterial infections, oxidative and DNA-damaging agents, UV light, and osmotic shock)), where the transcription factor binds to promoter regions of genes encoding proinflammatory inducible enzymes such as COX-2, iNOS, and other inflammatory-related proteins [44, 45]. These anti-inflammatory effects of Resv have been also observed in a variety of cell lines, such as HeLa cells, Jurkat, RAW 264.7 macrophage, or U-937 cells [46, 47], and in* in vivo* experiments in rodents [48].

In addition to suppressing LPS-induced NF-*κ*B-dependent COX-2 activation, Resv also activates AMPK [42, 47, 49] which effectively prevents tumorigenesis [29]. Thus, these mechanisms, at least in part, support the chemopreventive role of this stilbene.

On the other hand, studies on the redox status and functionality of the antioxidant machinery show the ability of Resv as a potent chemoprotector in different* in vivo* models of cancer development. Administration to rats of the potent hepatotoxic carcinogen azoxymethane (AOM) induced a potent oxidative unbalance triggered by glutathione (GSH) depletion, lipid peroxidation, and increased NO levels in the liver. All these effects were partially reversed by Resv administration [50]. Moreover, Resv acts as an antioxidant, at nutritionally relevant concentrations, by inducing the expression of superoxide dismutase (SOD) and catalase through a mechanism involving phosphatase and tensin homologue (PTEN)/protein kinase B (PKB) signaling pathway [51]. PTEN is a tumor suppressor gene and its expression is commonly decreased or lost in a large number of cancers of high frequency. The protein encoded by this gene is a phosphatidylinositol-3,4,5-trisphosphate 3-phosphatase and its main role is to dephosphorylate phosphoinositide substrates. So, it negatively regulates intracellular levels of phosphatidylinositol-3,4,5-trisphosphate in cells and acts as a tumor suppressor by negatively regulating the PKB signaling pathway. Inhibition of phosphatidylinositol 3-kinase (PI3K)/PKB pathway by PTEN has been associated with upregulation of SOD, GSH peroxidase, and catalase activities [52]. We confirmed these antioxidant properties of Resv in a mouse model. The pretreatment of mouse skin with Resv decreased several ultraviolet B radiation- (UVB-) induced oxidative events in a dose-dependent manner. Resv administration restored GSH levels, SOD, GSH peroxidase, and catalase activities to control values (mice without UVB irradiation) [53].

Despite scientific advances regarding the biological effects of Resv, our understanding of its anticancer mechanisms is far from a complete understanding. There are numerous evidences showing the capability of this polyphenol to induce programed cell death in different types of cancer. Proapoptotic stimulation by Resv has been associated with cell cycle alterations [54–56], caspase induction [54, 55, 57, 58], downregulation of Bcl-2, Bcl-xL, Survivin, and XIAP levels [59], and upregulation of Bax levels [58, 59], Bak, PUMA, Noxa, P21, Bim, TRAIL-R1/DR4, and TRAIL-R2/DR5 [59, 60]. Interestingly, a number of these effects may be correlated with P53 activation [55, 57–59]. For instance, Resv and piceatannol increased the cytoplasmic concentration of calcium in MDA-MB-231 human breast cancer cells, which induced the activation of P53 and the transcription of different proapoptotic genes [60]. Moreover, treatment of mutant P53 prostate cancer DU145 cells with Resv induced phosphorylation of the tumor suppressor which restored wild-type P53 DNA binding [61, 62] and P53 acetylation [63], activating proapoptotic events.

### 2.2. Pterostilbene

Pterostilbene (3,5-dimethoxy-4′-hydroxystilbene; Pter) is a natural analog of Resv, but with higher bioavailability [64, 65]. Due to its close structural similarity Pter possesses significant antioxidant activity* in vitro* in comparison with Resv [66, 67] and a clear clinical potential in different diseases [68]. Moreover, Pter has been reported to have cancer chemopreventive properties in different* in vitro* and* in vivo* experiments and other Resv-like health benefits. In these experiments, Pter was shown to inhibit growth, adhesion, and metastatic growth and to be an active apoptotic agent [68–71]. These effects have been shown in different types of cancers such as breast cancer [54, 68, 72–74], lung cancer [54, 75, 76], stomach cancer [68], prostate cancer [77], pancreatic cancer [78], melanoma [54], or colon carcinoma [54].

As it occurs with Resv, the antioxidant properties of Pter may also contribute to cancer chemoprevention. Rimando et al. [66] demonstrated that the antioxidant activity of Pter inhibits carcinogen-induced preneoplastic lesions in a mouse mammary organ culture model. Later, Chiou et al. [79] demonstrated that Pter is more potent than Resv in preventing AOM-induced colon tumorigenesis via activation of the Nrf2-mediated antioxidant signaling pathway. In the same experimental model, Pter decreased the expression of inflammatory genes, such as iNOS and COX-2 [80, 81]. Similarly, in HaCaT immortalized human keratinocytes Pter increased Nrf2 translocation into the nucleus and expression of Nrf2-dependent (oxidative stress related) molecules, thus further supporting the role of Nrf2 as a central regulator in the chemoprevention effect elicited by Pter [53]. Furthermore, in cultured HT-29 colon cancer cells the cytokine induction of the p38-activating transcription factor 2 pathway was markedly inhibited by the polyphenol compared to other anti-inflammatory pathways, such as NF-*κ*B, Janus-activated kinase-signal transducer and activator of transcription (JAK-STAT), extracellular signal-regulated kinase (ERK), c-Jun NH2-terminal kinase, and PI3K [80]. That inhibition was associated with iNOS and COX-2 reduction, suggesting that p38 mitogen-activated protein kinase cascade is a key signal transduction pathway for the anti-inflammatory action of Pter [80].

Pter has also been found as potent as Resv in inhibiting NF-*κ*B, AP-1, COX-2, and iNOS in a 12-O-tetradecanoylphorbol-13-acetate (TPA) induced mouse skin carcinogenesis model [82]. Moreover, Pter induces the expression of PTEN in prostate cancer decreasing the levels of miR-17, miR-20a, and miR-106b. The effect in restoring both PTEN mRNA and protein levels was lower for Resv [83], thus suggesting that Pter might show higher* in vivo* activity due to the substitution of hydroxyl by methoxy groups. In this context, we have recently published that, in an UVB-induced mouse skin carcinogenesis model, Pter is clearly superior to Resv in preventing acute and chronic skin damage [53]. In this study we demonstrated that the anticarcinogenic effect associated with a Pter-induced maintenance of skin antioxidant defenses (i.e., GSH levels, catalase, superoxide, and GSH peroxidase activities) and a reduction of UVB-induced oxidative damage on proteins, DNA, and lipids [53].

In addition, numerous studies have corroborated that Pter is an efficient anticancer agent acting on multiple signal transduction pathways. In the AOM-induced colon carcinogenesis model in rats, Pter, administered in the diet, decreased formation of aberrant crypt foci [81, 84]; transcriptional activation of iNOS and COX-2; GSK-3b phosphorylation and Wnt/b-catenin signaling; expression of VEGF, cyclin D1, and MMPs; activation of Ras, PI3K/PKB, and EGFR signaling pathways [84]; and mucosal levels of the proinflammatory cytokines, TNF-*α*, IL-1b, and IL-4 [85] and reduced the nuclear presence of phospho-p65 [85]. Moreover, McCormack et al. [86] showed the inhibitory effect of Pter on leptin-stimulated breast cancer* in vitro* through reduction of cell proliferation and JAK/STAT3 signaling. After that, microarray analysis of Pter-treated pancreatic cancer cells revealed upregulation of proapoptosis genes and altered levels of phosphorylated STAT3, MnSOD antioxidant activity, cytochrome C, and Smac/DIABLO [78]. Moreover Liu et al. have recently reported the ability of Pter to inhibit JAK2/STAT3 signaling downregulating the expression of STAT3 target genes, including the antiapoptotic proteins Bcl-xL and Mcl-1, and leading to upregulation of mitochondrial apoptosis pathway-related proteins (Bax, Bak, cytosolic cytochrome c, and cleaved caspase 3) and cyclin-dependent kinase inhibitors such as p21 and p27 in osteosarcoma [87].

The chemopreventive role of Pter is not limited to its antioxidant and anti-inflammatory properties or the cell death induction by apoptosis. It has been suggested that this stilbene may induce cell death, also, by autophagy [73, 76, 77, 88, 89]. However, the initial observations were based on accumulation of LC3II and autophagosomes, which is not a clear evidence of autophagic cell death [90, 91]. In fact, autophagosomes and LC3II accumulation are not significantly associated with active autophagy [54, 92]. Recently we have shown that Pter-induced tumor autophagy is an hsp70-dependent lysosomal membrane permeabilization mechanism [54].

The traditional cancer progression model has been rewritten in the last years highlighting the importance of tumor heterogeneity in chemo/radio-resistance development and relapse after treatment. In this sense, cancer stem cells (CSC) have emerged as a highly tumorigenic cell pool displaying properties of normal stem cells such as their ability to self-renew, to form differentiated progeny, and to generate a heterogeneous lineage of all types of cancer cells within a tumor, thus turning into a very attractive anticancer target [93–95]. In this context, it has been described that Pter and Resv can promote expression and activity of Argonaute-2, a central RNA interference (RNAi) component, which inhibits breast cancer stem-like cell characteristics by increasing the expression of a number of tumor-suppressive miRNAs (including miR-16, miR-141, miR-143, and miR-200c) [96]. Pter suppressed not only the generation of CSC but the metastatic potential in different experimental models [97, 98]. Under the influence of tumor-associated macrophages, which promote tumor growth and progression, Pter was shown to modulate epithelial-to-mesenchymal transition signaling pathways [97]. In addition, this stilbene was able to prevent the enrichment of CD133(+) hepatoma CSCs under irradiation [98].

### 2.3. Piceatannol and Pinosylvin

Piceatannol (trans-3,5,3′,4′-tetrahydroxystilbene) is a hydroxylated analog of Resv found in a variety of plant sources including, for example, grapes, peanut, passion fruit, and white tea. Although less studied, piceatannol has health-promoting effects similar to Resv [99–101]. Li et al. [102] showed that the anticancer properties of piceatannol may be attributed to its prooxidant properties, which in the presence of copper (Cu)(II) induces formation of the hydroxyl radical through the Haber Weiss and Fenton reactions and DNA breakage. In fact, there are authors that propose that the anticancer action of plant polyphenols involves, in part, mobilization of endogenous copper and its consequent prooxidant action [103].

Paradoxically, in accordance with its origin and structure, piceatannol also shows similar activities as those indicated for Resv such as the antioxidant activity, although mediated by different pathways. Piceatannol inhibits NF-*κ*B activation by H_2_O_2_, phorbol 12-myristate 13-acetate, LPS, okadaic acid, and ceramide [104]. Moreover, piceatannol inhibits TNF-induced I*κ*-Ba phosphorylation, p65 phosphorylation, and p65 nuclear translocation. These effects, also observed under Resv treatment, suggest a crucial role of the hydroxyl group in positions 3 and 4′ [104]. It has also been described that piceatannol reduces the expression of iNOS, decreasing the NO production, and COX-2 in LPS-stimulated RAW 264.7 cells and BV2 microglia cells [105, 106]. Piceatannol is also able to increase heme oxygenase-1 expression and protein levels in human breast epithelial MCF10A cells. The underlying mechanism involves stimulation of Nrf-2 release from Keap1, nucleus translocation, and direct binding of the transcriptional factor to the antioxidant response element, leading to an enhancement of heme oxygenase-1 expression [107].

Regarding pinosylvin (3,5-dihydroxy-trans-stilbene), a pine antifungal and antibacterial stilbene, its chemopreventive activity may be also attributed to its antioxidant and anti-inflammatory activity. In fact, pinosylvin, like Resv or piceatannol, inhibited the production of PGE_2_ in LPS-induced RAW 264.7 cells, thereby inhibiting the expression of COX-2 [108]. Later, in the same cellular model it was shown that pinosylvin is also able to inhibit iNOS expression [109].

## 3. *In Vivo* Toxicity of Resveratrol and Pterostilbene

### 3.1. Resveratrol

An initial starting point in the safety evaluation of a naturally occurring food substance is its natural intake. The daily intake of dietary Resv is mainly from the consumption of wine and grapes and foods derived from them. This intake of up to 2 mg/day is relatively low in comparison to the level of safe oral intake that is derived from oral preclinical studies with ResVida, a high purity trans-Resv formulation [110]. This compound, commercialized by DSM Nutritional Products Ltd., obtained GRAS (Generally Regarded As Safe) designation by the U.S. Food and Drug Administration (FDA) in 2008, with his Allowable Daily Intake (ADI) being 450 mg/day [110]. The ADI was based on no-observed-adverse-effect-levels (NOAELs) of 750 mg/kg bw/day in rats on a 13-week developmental toxicity study by the dietary route and a standard safety margin of 100 [111]. Although it has been also described, in studies by gavage, that Resv caused toxicity in the kidney and bladder after 4-week treatment in rats, this was at very high dosages (2.000–3.000 mg/kg bw/day) [112].

Regarding Resv's toxicity versus time, six-month studies in rat and rabbit models showed no significant increase in toxicity in comparison to the 4-week studies [110]. Kinetic data from the DSM 13-week toxicity study support the expectation of no increase in toxicity with longer term intake [111]. About Resv genotoxic activity, short-term studies based on the Ames test showed that this compound does not have genotoxic activity* in vivo*, but experimental details are too limited to evaluate the data in full [111].

Only a small number of clinical trials using Resv as a single-agent, and formulated as a medicinal product, have formally addressed and reported on safety and tolerability [113–117]. No serious adverse event was detected in all these studies. Adverse events were mild and only lasted for a few days. The most common toxicity was gastrointestinal, particularly diarrhea, nausea, and abdominal pain, but also frontal headache and rash occurred in some patients. A sequential dose study of Resv at repeated daily doses of up to 5 g (0.5, 1.0, 2.5, and 5.0 g) for 29 days in healthy volunteers was performed. The results of these clinical, biochemical, and hematological analyses showed that Resv administration is safe, although at the 2.5 g and 5 g dose levels it caused reversible gastrointestinal symptoms such as diarrhea, nausea, or flatulence in some individuals.

It is worthwhile to mention that a phase II clinical trial (https://www.clinicaltrials.gov/), sponsored by GlaxoSmithKline in patients with multiple myeloma to assess the safety and activity of SRT501 (a micronized formulation of Resv), was terminated due to safety concerns after kidney damage (cast nephropathy) developed in some patients. In this trial, a high dose of 5 g SRT501/day was administered orally for 20 consecutive days. This dose of Resv was significantly higher than that used in the safety study mentioned above for ResVida [110]. Nevertheless cast nephropathy is a condition closely associated with multiple myeloma, so the finding in this study is of doubtful significance outside of this disease condition.

### 3.2. Pterostilbene

The toxicity of Pter, after intravenous administration to xenografted mice, has been assessed in several studies involving the treatment of colorectal cancer [118], prostate cancer [119], and melanoma [69]. The doses and time of administration were 20 mg/kg and 30 mg/kg per day during 23 days [118]; 50 mg/kg per day during 4 weeks [119]; and 20 mg/kg during 10 days [69]. In all these studies, Pter was found therapeutically effective and pharmacologically safe because it showed no organ-specific or systemic toxicity.

Regarding oral administration, Ruiz et al. [120] published in 2009 a study in which they evaluated the toxicity of Pter at high doses in healthy mice. For this purpose, mice were fed during 28 days at doses of 30, 300, and 3000 mg/kg body weight/day of Pter. These daily doses did not cause mortality during the experimental period at any dose, but the red blood cell number and hematocrit increased after Pter administration compared to control groups. However, histopathological examination and evaluation of biochemical parameters revealed no alterations regarding clinical signs or organ weight at any dose [120].

Chromadex Inc. (Irvine, CA) achieved GRAS status for its ingredient pTeroPure-branded Pter (http://www.fda.gov/) in 2011. The ADI for pTeroPure is up to 30 mg/kg per day for food use (https://chromadex.com/NewsEventDetail.aspx?Aid=510). Data from the first clinical trial on Pter (Effect of Pter on Cholesterol, Blood Pressure and Oxidative Stress, https://www.clinicaltrials.gov/, conducted at the University of Mississippi Medical Center) were released in 2012. It was concluded that oral administration of 125 mg of Pter twice per day was well-tolerated because there were no statistically significant adverse drug reactions on hepatic, renal, or glucose markers based on biochemical analysis [121]. Despite these observations, more rigorous studies are needed before dietary/therapeutic dosages can be standardized for different applications.

## 4. Pharmacokinetics of Stilbenes

Stilbenes, as the majority of phenolic compounds, have low bioavailability which limits their potential benefits for health [122]. The bioavailability depends on the route of administration but also relies on their absorption and metabolism. Those factors are mainly determined by the chemical structure of the compound (degree of glycosylation/acylation, their basic structure, conjugation with other phenolics, molecular size, degree of polymerization, solubility, etc.) [11, 123]. That is the reason why bioavailability may greatly differ among the many different (even closely related) phenolic compounds.

### 4.1. Resveratrol

Many concerns regarding Resv effectiveness* in vivo* arise from its low bioavailability and short half-life. According to Asensi et al. [124], after intravenous administration to rabbits of 20 mg of Resv/Kg its highest concentration in plasma was 42.8 ± 4.4 *μ*M 5 min after administration. But, because of its rapid metabolism and short half-life (14.4 min), this concentration decreased very rapidly at 60 min to 0.9 ± 0.2 *μ*M. After oral administration of the same dose the highest concentration in plasma within the first 5 min was lower (2-3 *μ*M), thus indicating the higher limitations on bioavailability linked to the oral intake. Similar results were reported by others in humans [125, 126].

**Figure 2:**
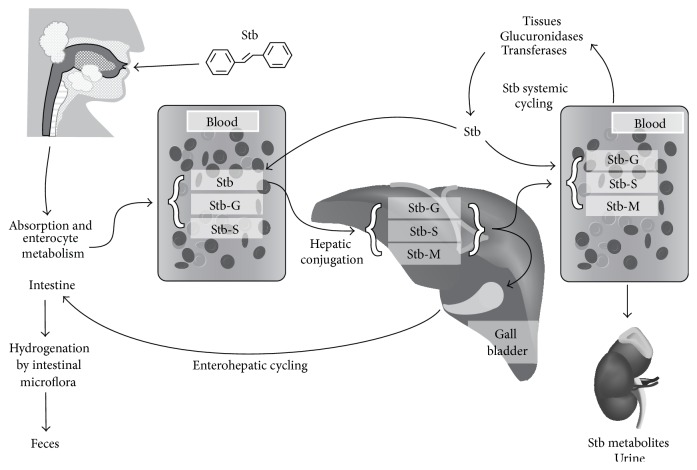
General metabolic pathways of the major stilbenes.

However Resv is highly absorbed after oral administration (about 75% of the dose administered to humans) mainly by transepithelial diffusion [127]. Therefore, its low bioavailability is caused by its rapid and extensive first-pass metabolism in the intestine and liver. Once metabolized, Resv is excreted through the urine and feces although some conjugated metabolites can be also reabsorbed by enterohepatic recirculation (Figure 2) [126, 128].

The main metabolites of Resv are produced through three metabolic pathways: glucuronic and sulfate conjugation of the 3 and 4′ phenolic groups (phase II metabolites) and hydrogenation of the aliphatic double bound. The latter has been suggested to be produced by intestinal microflora [126, 127, 129] (Figure 2). Up to nearly 20 Resv-derived metabolites have been described in plasma, urine, and some tissues [115, 126, 130–134]. Among these metabolites there are mono- and diglucuronides; monosulfates, disulfates and trisulfates; and sulfoglucuronides, as well as equivalent conjugations of the hydrogenated Resv.

In plasma, the major circulating metabolites of Resv are phase II conjugates, being the most abundant Resv-3-sulfate in humans [115, 116, 131]. In contrast, in rats and pigs is Resv-3-glucuronide the main metabolite. In both cases the plasma concentrations of Resv metabolites are much higher than the concentration of the parent molecule [128, 135].

The efficacy of the Resv metabolites is still under debate. Emerging data suggests that Resv conjugates have anticancer activity* in vitro*. The biological effects of those metabolites appear to be reduced when compared to Resv in some studies [136, 137] and similar in others [138–140]. However, although Resv glucuronides have some biological effects, no cytotoxic activity against cancer cell lines has been demonstrated. Only one study has reported cytotoxic activity of glucuronide metabolites but only when administered as a mixture of them [141]. Nevertheless, a common hypothesis is that as it has been reported for other compounds [142, 143], these metabolites could undergo deconjugation, releasing the parent compound (Figure 2). Consequently, the glucuronide and sulfate conjugates of Resv may provide a pool from which active Resv can be released [129]. This hypothesis has been proved recently for Resv sulfate conjugates in mouse [144] although it is uncertain (very unlikely in fact) that Resv deconjugation may release sufficient effective levels, in terms of real biological activity, under* in vivo* conditions.

The biological activity of the Resv metabolite dihydroResv is also incompletely understood. In some* in vitro* studies it exerts an antiproliferative effect in tumor and normal cell lines but less potent than Resv [67, 139]. On the other hand, a recent study* in vitro* shows potent antiproliferative effects on hormone-sensitive breast cancer cells [145]. However, since the dihydroResv metabolite is mainly formed in the colon, it might be expected that it contributes to chemopreventive effects at that site [127]. In fact, sulfate and glucuronide conjugates of dihydroResv have been found in cecum, colon, and rectum of the pig [130] and in the colon of the rat [129].

Recently, the detection of Resv and its derived metabolites has been reported in colorectal tissue of patients after oral treatment with Resv [117]. The major metabolites found in tumor and normal tissue were phase II metabolites (glucuronides, sulfates, and sulfoglucuronides). The highest concentration was detected for Resv-sulfoglucuronide. However, the possible concurrency of dihydroResv and derived conjugates was not explored [117].

### 4.2. Pterostilbene

Pter, as a natural occurring dimethoxy analog of Resv, has a more favorable pharmacokinetics profile [64, 65, 70]. On one hand, as Pter has less hydroxyl groups (only one instead of three in Resv), it is less susceptible to conjugation metabolism and, therefore, is predicted to have a longer half-life [146, 147]. On the other hand, the dimethoxy structure enhances its lipophilicity thus increasing membrane permeability and improving its bioavailability [64, 146, 148].

Similarly to Resv, the major Pter metabolites found in mouse plasma and urine are phase II conjugates: Pter glucuronide, Pter sulfate, monodemethylated Pter glucuronide, monodemethylated Pter sulfate, monohydroxylated Pter, monohydroxylated Pter glucuronide, monohydroxylated Pter sulfate, and monohydroxylated Pter glucuronide sulfate [64, 70, 149]. Nevertheless, there is no evidence of the presence of a Pter hydrogenated form or the equivalent phase II conjugations of this. Those metabolites have been reported to be recycled by enterohepatic recirculation as it has previously been reported for Resv (Figure 2) [70]. No studies are available at the moment on the possible biological activity of Pter metabolites.

After intravenous administration in mouse of Pter and Resv, either of them reaches their highest concentrations within the first 5 minutes. However, while for Resv this concentration decreased very rapidly to 1 *μ*M within the first 60 minutes, Pter remains longer in plasma reaching the 1 *μ*M concentration in 480 minutes [69, 124]. From these data, the calculated half-life for Pter is 6-7 times longer than for Resv [69, 124]. Similar results have been reported in recent studies in rats [65, 70].

Regarding oral bioavailability, it has been reported that in rats it is greater for Pter (80%) than for Resv (20%) [64]. Also in this study, as it has been reported by others [150], the major metabolite in plasma is Pter sulfate, with its levels being higher than those of the parent compound. In addition, plasma levels of Pter and Pter sulfate after oral administration were greater than Resv and Resv sulfate, respectively, whereas levels of Resv glucuronide were higher than Pter glucuronide. Another issue to point out is that after Pter administration Resv was not detectable, indicating that Pter is not a prodrug of Resv [64].

Interestingly, a recent study reports that levels of Pter and its main metabolite, Pter sulfate, are higher in tissues than in blood, meaning that they accumulate in tissues where Pter conjugates may act as a source of the natural compound. This observation has logical implications for the* in vivo* bioactivity of Pter because it may explain the paradox of Pter biological activity despite its low plasma concentrations [150]. Levels of Pter sulfate were higher than levels of Pter in every organ except the brain, where levels of the parent compound were higher. This observation has a particular interest given the reports of the biological activity of Pter on the central nervous system [150].

### 4.3. Piceatannol and Pinosylvin

These stilbenes, after intravenous administration in rats, are distributed into tissues and highly extracted by the liver where they undergo extensive glucuronidation. All are predominantly eliminated via nonurinary routes and as they have short half-lives, their estimate oral bioavailability is poor as it is the case for Resv and Pter [151–155].

The major metabolic pathways for piceatannol are glucuronidation and sulfation, as it occurs for Resv and Pter, but also methylation [153]. In contrast to piceatannol, methylated metabolites have not been found in rat plasma after treatment with Resv [153, 156]. Piceatannol conjugates could be also recycled by enterohepatic recirculation as the other stilbenes (Figure 2) [157].

A remarkable finding is that piceatannol can be metabolized into another stilbene, the isorhapontigenin, suggesting that piceatannol could exhibit additional biological functions [153]. Compared to Resv piceatannol may have a higher metabolic stability and similar beneficial effects [100, 101, 153]. In fact, it has been suggested that anticancer properties of Resv may be due to its metabolism to piceatannol by the cytochrome P450 enzyme CYP1B1, suggesting that Resv may act as a source or prodrug of piceatannol [158].

Glucuronidation has been described as the major conjugation pathway of pinosylvin. However, interestingly, two minor oxidized metabolites of this polyphenol have been detected: Z- and E-Resv [151, 154]. Structurally, pinosylvin, compared to Resv, lacks the 4′-hydroxyl group while it retains the 3-hydroxyl moiety which has been identified as a major target of phase II conjugation reactions. Furthermore, the absence of the 4′-hydroxyl group in pinosylvin may have enhanced its binding to first-pass metabolic enzymes. Thus, due to its extensive metabolism, compared to Resv, pinosylvin has lower oral bioavailability [155].

## 5. Analyses of Structure-Activity Relationships to Improve the Effectiveness of Stilbenes

Generally, the main problem regarding the use of polyphenols is the partial knowledge of their mechanisms of action [159] and their low bioavailability [124], which as mentioned before is determined by their chemical structure [160–162]. These features regulate both absorption and excretion of phenolic compounds. As an example, 0.3% of the intake of anthocyanins is excreted by urine, compared to 43% of isoflavones, thus reflecting the potential importance of the chemical structure [163].

There are a high number of works in which the structure-activity relationships (SARs) of polyphenols are studied. These studies intend to figure out, using structural analogs, which modifications may confer increased resistance to oxidation of the polyphenols [164], improving the interaction with domains of the target proteins [159] and finally increasing the pharmacokinetics properties [165]. Theoretically, part of these changes may help to direct certain polyphenols to target tissues [166, 167].

The main changes in structural analogs affect the number and position of hydroxylated and methylated groups, which also influence their metabolism. In fact, polyphenols metabolized to their secondary metabolites may even have more activity. However, there are critical residues for the functional groups that are directly linked to the activity; for example, hydroxylation at C4 in Resv analogues is critical to its function in* in vitro* studies [165]. Structure-activity studies have revealed that increasing the number of OH groups at their ortho position on the phenol ring of stilbenes could increase the free radical scavenging capacity, the cytotoxic activity, and the anti-inflammatory effects of these compounds [100, 168]. In fact, polyhydroxylated analogs of Resv as hexahydroxystilbene turned out to be more potent and specific inhibitors of COX-2 activity than Resv both* in vivo* and* in vitro* [168, 169]. Moreover, this analog, showing higher antiradical activity, also induces apoptosis at concentrations than the parent compound [168].

Nevertheless, in animal studies, the 3,4,5,4′-tetramethoxystilbene (DMU-212), wherein the C4-OH is blocked by methylation, possesses stronger antiproliferative properties in human colon cancer cells than Resv, possibly, because these methylated groups, by slowing excretion, could provide better plasma levels [159]. Another example is pinosylvin. Pinosylvin differs from Resv in lacking one hydroxyl at C4′, which makes it more lipophilic but losing its antioxidant activity. Nevertheless, once inside the cell, it recovers the antioxidant activity [170]. The methoxylated analogs have higher lipophilicity, which may favor their entry into cells and confer more resistance to degradation, thus improving pharmacokinetics [54, 171]. However, the number of methoxy and hydroxyl groups must be under equilibrium. The hydroxyl groups confer more solubility, which allows a better interaction with proteins [166], whereas the methoxylated group confer resistance to degradation although an excessive number of methoxylated groups may impair the interaction with the target protein [165]. Pter, with two methyl groups, a trans-3,4′-dihydroxy-2′,3′,5-trimethoxystilbene with higher anticancer effects than Resv both* in vitro* and* in vivo* [54, 172].

Polyphenols exhibit excellent healthy and therapeutic properties to treat various diseases, including a broad spectrum of actions involved in a large number of targets and very low toxicity properties. Manipulation of the polyphenolic structure can improve its bioavailability and activity. DMU-212 is an example of a more lipophilic structural analog of Resv capable of crossing the blood-brain barrier [173]. These successes show that modifying the polyphenolic structures we may be able to exploit their properties improving its activity and action.

## 6. Clinical Trials

Natural stilbenes have been used in traditional medicine. Resv, piceatannol, and Pter are examples of stilbenes synthesized by several types of plants in response to a variety of stress conditions [79]. Starting on their implication on the known “French paradox” (which associates red wine consumption and lower coronary heart disease), several clinical and pharmacometric studies on Resv have been performed in the last years. Even though most pharmacometric studies of Resv in humans show that its plasma concentrations are below the effectiveness range indicated by* in vitro* assays, it may show interesting effects* in vivo*. Boocock et al. [115] found out that administration of a single dose of Resv (5.0 g) rendered a peak plasma concentration of 2.4 nmol/mL, which is only slightly below the required concentration* in vitro* to show chemopreventive properties. Most clinical studies on Resv and cancer have been performed in colorectal cancer patients, possibly because oral administration may facilitate reaching higher concentrations of this stilbene in tumors located along the gastrointestinal tract. For example, a clinical assay by Patel et al. showed that 0.5 g and 1.0 g doses of Resv were able to significantly reduce colorectal cell proliferation [117]. Howells et al. [174] assayed micronized Resv SRT501 in colorectal cancer patients with hepatic metastases, who had not received therapeutic intervention for their cancer within 6 weeks of study commencement and had a life expectancy of less than 3 months. They concluded that SRT501 administration during 21 days was safe, although some individuals suffered some adverse effects like nausea or diarrhea. The study accomplished by Noguer et al. [175] showed that alcohol-free red wine consumption can increase our antioxidant enzyme activities (SOD, catalase, and GSH reductase). This assay demonstrated that alcohol-free red wine may improve the health of people suffering oxidative stress related diseases.

At present, despite the promising anticancer properties elicited by Pter, there is only a clinical trial performed at the University of Mississippi. In this clinical trial researchers assessed the effects of Pter in cholesterol, blood pressure, and oxidative stress. They showed that Pter is able to improve these aforementioned parameters under safe conditions (ClinicalTrials.gov Identifier NCT01267227). Clinical trials on specific anticancer effects are expected to be performed in the next future.

## 7. Conclusions

The identification of protective molecules without side effects should be a main objective in the fight against cancer. Experimental* in vitro* and* in vivo* studies, and a few clinical trials, show evidences about the effectivity of stilbenes as anticancer agents, both in the form of nutritional supplements or functional foods and as potential anticancer drugs. This group of polyphenols show a very low toxicity and, although having multiple molecular targets, act on different protective and common pathways usually altered in a great number of tumors. This is important since it suggests that natural stilbenes may be more prone for their use as anticarcinogens. The capability to prevent carcinogenesis includes inhibition of inflammation, oxidative stress, and cancer cell proliferation and using tightly regulated cell death mechanisms. Due to the complexity and number of cellular processes involved more studies must be done to fully understand how stilbenes may be used to avoid the development of cancer. Moreover, due to their low concentration in food and their rapid metabolism and excretion in the body mammals, improvements in delivery systems, stability, and solubility are necessary in order to make their use in clinical settings as chemopreventive drugs possible.
